# Prognostic significance of miR-203 and ZEB1 expression in early-stage hepatocellular carcinoma

**DOI:** 10.7150/jca.57819

**Published:** 2021-06-11

**Authors:** Hongyuan Chen, Meng Kong, Ying Chen, Yugang Jiang, Mingxin Wen, Xinke Zhang

**Affiliations:** 1 Department of General Surgery, Shandong Provincial Hospital Affiliated to Shandong First Medical University, Jinan, Shandong 250021 China.; 2 Department of Gynaecology, People' Hospital of Rizhao, Rizhao, Shandong 276800 China.; 3 Department of Human Anatomy and Key Laboratory of Experimental Teratology, School of Medicine, Shandong University, Jinan, Shandong 250012 China.; 4 Key Laboratory of Chemical Biology (Ministry of Education), Department of Pharmacology, School of Pharmaceutical Sciences, Cheeloo College of Medicine, Shandong University, Jinan, Shandong 250012 China.

**Keywords:** miR-203, Hepatocellular carcinoma, ZEB1, Prognosis

## Abstract

**Background:** Approximately one-quarter of patients with early-stage hepatocellular carcinoma (HCC) suffer from tumor recurrence within the first year after hepatectomy. Identification of patients at high risk of recurrence and new therapeutic approaches are crucial to improve clinical outcome. This study aimed to assess the prognostic significance of miR-203 and Zinc finger E-box binding homeobox 1 (ZEB1) in early-stage HCC and explore the association between the expression of ZEB1 and miR-203 in HCC.

**Methods**: Tissue microarray-based immunohistochemistry (IHC) and *in situ* hybridization (ISH) were performed to investigate ZEB1 and miR-203 expression in 73 patients with early-stage HCC and their correlation with clinicopathological features and prognosis of patients were analyzed. The prognostic value of the two factors was also measured by public KM plotter database. Quantitative reverse transcription PCR (qRT-PCR) assays were conducted to study the relationship between miR-203 and ZEB1. Transwell assays, Cell Counting Kit-8 (CCK-8) assays were performed to detect the roles of miR-203 in migration, invasion and proliferation of HCC cells.

**Results:** We found low expression of miR-203 was associated significantly with tumor recurrence (*P*<0.001) and poor survival (*P*=0.020) of patients with early-stage HCC. Multivariate analysis revealed that low miR-203 expression was a poor prognostic factor for both overall survival (OS) (*P*=0.036) and recurrence free survival (RFS) (*P*=0.017). ZEB1 did not show any prognostic significance in our cohort. Correlation analysis indicated that there was no significant correlation between miR-203 and ZEB1 on both mRNA and protein levels. Furthermore, functional studies indicated that miR-203 repressed migration, invasion and proliferation of HCC cells *in vitro*.

**Conclusion**: Our study suggested that miR-203 could be a novel predictor in early-stage HCC and might also be a potential molecular target for HCC therapy.

## Introduction

Hepatocellular carcinoma (HCC) is the most common primary cancer of the liver and the fourth leading cause of cancer-related death worldwide 1. Despite many advances in diagnosis and treatment, HCC patients still have a dismal prognosis 2-4. The prognosis of patients with HCC largely depends on their tumor stage because curative treatments are available only for patients with early-stage disease 5. Liver transplant (LT) is considered the most effective method to treat both the cancer and the liver disease underlying most cases of HCC 6, but it is not readily available to everyone mainly because of tumor stage and limited donor availability. Liver resection remains the gold standard for patients with resectable HCC. However, approximately one-quarter of patients with early-stage HCC [the Barcelona Clinic Liver Cancer (BCLC) stage 0/A] suffer from tumor relapse within the first year after surgical resection 7. The distinct outcomes may be attributed to tumor heterogeneity. Hence, molecular investigation of HCC could provide information for predicting recurrence and new therapeutic targets.

Using microarray analysis and TaqMan real-time PCR, we previously found that miR-203 expression was lower in the tumor tissue of patients with post-LT HCC recurrence than in that of patients without recurrence. Patients with higher miR-203 expression had a significantly better prognosis than patients with lower expression 8, 9. Because of whole liver removal, HCC recurrence after transplantation is considered as metastasis-related recurrence. We considered that miR-203 may play an important role in HCC matastasis. However, its prognostic value and molecular mechanism in early-stage HCC are currently unclear.

Zinc finger E-box binding homeobox 1 (ZEB1) is a transcription factor that is physiologically involved in multiple processes, such as cell differentiation and tissue development 10. Recently, a growing amount of evidence has suggested that ZEB1 plays a central role in epithelial-to-mesenchymal transition (EMT) during tumor metastasis 11-13. In HCC, a few studies reported that high ZEB1 expression was correlated with malignant tumor progression and a poor prognosis 14-16. In non-small cell lung cancer (NSCLC) and gastric cancer (GC), ZEB1 was validated as a target of miR-203^17^, ^18^. More interestingly, ZEB1 was also reported to promote pancreatic cancer recurrence and metachronous metastasis after curative surgery and adjuvant chemotherapy by repressing miR-203 19, 20. However, whether the regulatory network of ZEB1/miR-203 is involved in HCC metastasis has yet to be determined.

In this study, we aimed to assess the prognostic significance of miR-203 and ZEB1 in early-stage HCC and explore the association between the expression of ZEB1 and miR-203 in HCC tissues. Subsequently, we completed a series of *in vitro* experiments to investigate the roles of miR-203 in HCC.

## Materials and methods

### Patients and tissue samples

A human HCC tissue microarray (TMA) that included HCC tissue samples and adjacent normal tissue samples from 90 patients was obtained from Shanghai Outdo Biotech Company (LivH180Su06, Shanghai, China). Inclusion criteria were as follows: (1) histologically diagnosed with HCC by pathologists; (2) Barcelona Clinic Liver Cancer (BCLC) stage 0/A; (3) solitary HCC; (4) detailed clinical histories and follow-up information; (5) without any previous treatment for HCC. Among the 90 HCC patients, 17 were excluded for one of the following reasons: (1) history of malignancy other than HCC; (2) multifocal HCC; (3) insufficient clinical information. Table 1 presents the detailed clinical parameters of the patients. Hepatitis B surface antigen (HBsAg) was determined by chemiluminescent microparticle immunoassay (CMIA) using Abbott Architect i2000SR analyser (Abbott Diagnostic, Chicago, IL, USA). Alpha-fetoprotein (AFP) level was performed by electrochemiluminescence immunoassay (ECLIA) using cobas e 601 immunoassay analyzer (Roche Diagnostics GmbH, Mannheim, Germany). The median time of follow-up was 53 months (range: 7-72 months), 40 patients had recurrent tumors, and 36 patients were dead at the end of the follow-up period. Recurrence-free survival (RFS) and overall survival (OS) times were calculated from the date of surgery to the date of tumor recurrence or death, respectively. All samples were collected with the informed consent of the patients, and the study was approved by the Institutional Review Boards of Shandong Provincial Hospital and was performed in accordance with the Declaration of Helsinki.

### *In situ* hybridization (ISH)

Locked nucleic acid (LNA) ISH analysis of the HCC TMA with a double DIG-labelled LNA probe specific for human miR-203 was performed according to the manufacturer's instructions (Exiqon, Denmark). In brief, the slides were deparaffinized in xylenes and rehydrated through an ethanol dilution series. The sections were then treated with Proteinase K at 37 °C for 10 minutes. After dehydration, the slides were incubated with 200 nM miR-203 probe at 50 °C for 1 h. Then, the slides were incubated with an alkaline phosphatase-conjugated anti-digoxigenin antibody (diluted 1:500, Roche) at 4°C overnight after stringency washes. Finally, hybridization signals were colourised with nitro blue tetrazolium (NBT) and 5-bromo-4-chloro-3-indolyl phosphate (BCIP) substrates. Stained samples were scanned using the Aperio ScanScope (Leica Biosystems) at 20× objective magnification.

The ISH analysis was evaluated by two independent pathologists in a blinded manner. A semiquantitative H-score was calculated by multiplying the staining intensity (0, negative; 1, weak; 2, moderate; and 3, strong) and the proportion of positively stained cells (0-100%; with any intensity of positive staining) for each sample 21.

### Immunohistochemical (IHC) staining

TMA sections were used for IHC staining. After deparaffinization, rehydration and antigen retrieval, the slides were incubated with an anti-ZEB1 antibody (1:100, ab203829, Abcam) at 4°C overnight. A Dako EnVision detection system (cat. K5007, DAKO, Denmark) was then employed to visualize the staining for ZEB1 following the manufacturer's instructions. Stained samples were scanned using the Aperio ScanScope (Leica Biosystems) at 20× objective magnification.

With respect to evaluation of IHC results, we referred to a previous method 15. ZEB1 expression was classified into four groups: 0(<1% positive cells), 1(1-5% positive cells), 2(6-10% positive cells), 3(>10% positive cells). ZEB1 positivity was defined as more than 1% positive nuclear staining.

### Public database KM-plotter analysis

Kaplan-Meier (KM) plotter database (http://kmplot.com) was used to explore the prognostic significance of miR-203 and ZEB1 in OS and RFS at the mRNA level. The database was established by use of microarray or RNA sequencing data with clinical information of HCC patients downloaded from Gene Expression Omnibus (GEO) and The Cancer Genome Atlas (TCGA) 22, 23. Briefly, miR-203 and ZEB1 were individually entered into the database and analysed with setting different clinical parameters. Then, Kaplan-Meier survival plots, hazard ratio (HR), 95% confidence intervals (CI) and log rank P were obtained on the webpage.

### Gene expression levels in human HCC cDNA array

Human HCC cDNA arrays (HlivH030PG01, Outdo Biotech, Shanghai, China) were used to determine miR-203 and ZEB1 mRNA levels by quantitative real-time PCR. The array contained normalized amounts of cDNA (2 ng normalized to actin) prepared from high quality tumors and adjacent normal tissues of 14 HCC patients. The expressions of miR-203 and ZEB1 were determined on a LightCycler 480 (Roche, Mannheim, Germany) according to the manufacturer's recommendation. The mRNA levels were normalized with actin, while miRNA levels were normalized with U6.

### Cell culture and transfection

The normal human liver cell line LO2 and four HCC cell lines (SMMC-7721, Hep3B, Bel7402, and PLC/PRF/5) were obtained from the Cell Bank of the Chinese Academy of Sciences (Shanghai, China). All cells were cultured in Dulbecco's modified Eagle's medium (DMEM; HyClone, Beijing, China) supplemented with 10% heat-inactivated foetal bovine serum (FBS; HyClone), 100 U/mL penicillin and 100 µg/mL streptomycin. Cells were cultured in an incubator at 37 °C in 5% CO_2_.

MiR-203 mimics and inhibitors and negative control oligonucleotides (GenePharma, Shanghai, China) were used to restore or inhibit miR-203 function. Transfection was performed using LipofectamineTM 2000 (Invitrogen) according to the manufacturer's instructions.

### Quantitative real-time PCR analysis

Total RNA was extracted from cells using TRIzol reagent (Invitrogen) following the manufacturer's protocol. For analysis of miR-203 expression, reverse transcription and PCR amplification were performed using TaqMan MicroRNA assays (Applied Biosystems). U6 snRNA expression was used as an internal control for quantification of miR-203 expression.

### Cell proliferation assay

Cell proliferation was evaluated using a Cell Counting Kit-8 (CCK-8; Dojindo, Kumamoto, Japan) assay according to the manufacturer's instructions. Briefly, 2×10^3^ cells were seeded in each well of 96-well plates. The cells were transfected with RNA oligonucleotides 24 h later and then cultured for 72 h before the addition of 10 μl of CCK-8 to each well. After a continued 2-h incubation, the absorbance was measured at 450 nm using the SpectraMax i3 Multi-Mode Microplate Detection Platform. Experiments were repeated independently 3 times.

### Cell migration and invasion assays

Migration and invasion assays were performed with noncoated and Matrigel-coated transwell chambers (24-well insert; pore size: 8 µm; Corning, NY, USA). In both assays, 8×10^4^ cells per well were placed in the upper chamber with serum-free DMEM, and culture medium containing 10% FBS was added to the lower chamber. The cells were incubated at 37°C for 24 (migration assay) or 48 h (invasion assay). The cells on the lower surface of the membrane were fixed in 4% paraformaldehyde and stained with 0.1% crystal violet dye, and five microscopic fields (at 200× magnification) were counted.

### Statistical analysis

Data analyses were performed using SPSS statistics version 20 software (IBM). The chi-square or Fisher exact test was used to determine the relationships between miR-203 or ZEB1 expression and clinical manifestations. Survival curves were plotted by the Kaplan-Meier method and compared by the log-rank test. Independent prognostic indicators were assessed by Cox regression with univariate and multivariate models. The Spearman rank correlation test was used to examine the association between ZEB1 and miR-203 expression in 73 HCC samples. Pearson' correlation analysis was performed between miR-203 expression and the mRNA levels of ZEB1. Data derived from *in vitro* experiments are presented as the mean ± SE and were assessed by a two-tailed Student's t test. *P* values <0.05 were considered to be statistically significant.

## Results

### Correlations between miR-203 expression and clinicopathological factors in early-stage HCC

**Figure 1:**
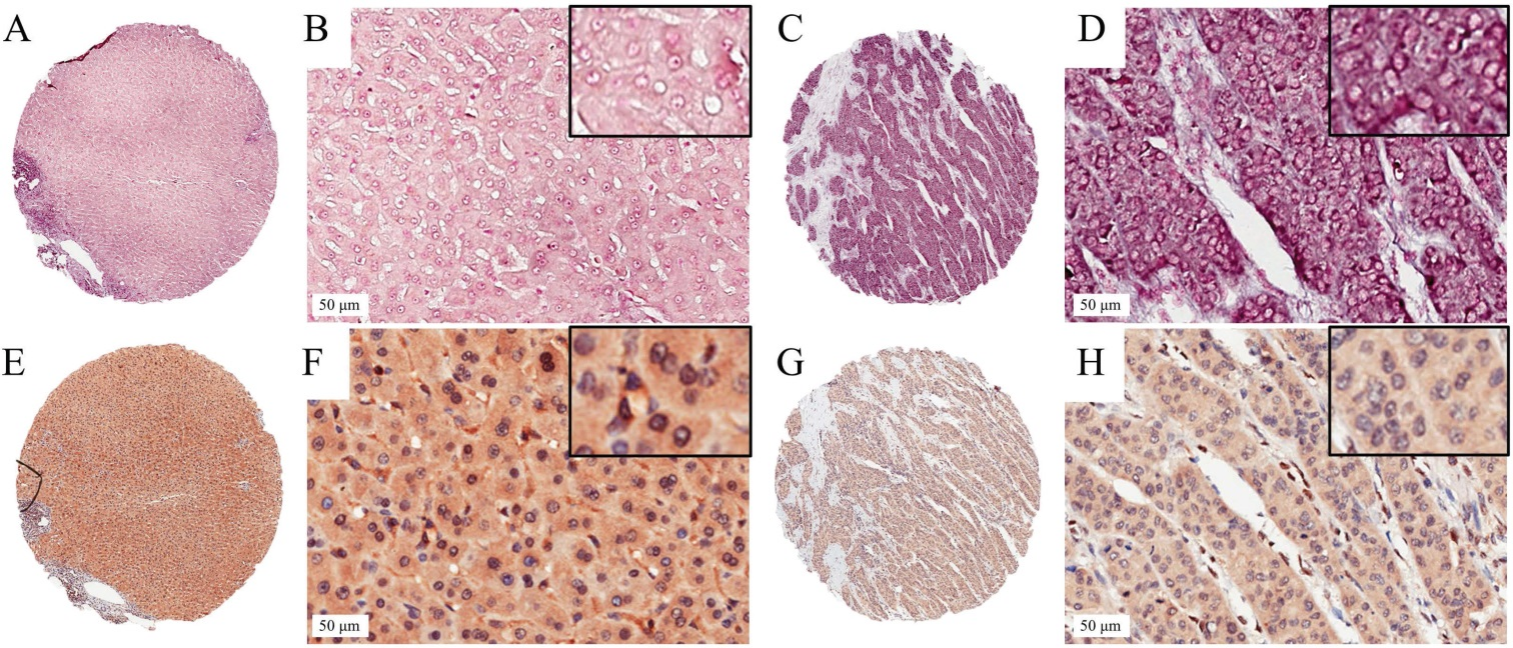
*In situ* hybridization for miR-203 and immunohistochemistry for ZEB1 in HCC TMA section. Representative images of miR-203 in HCC (A,B) and paired adjacent liver tissues (C,D). The miR-203 ISH signal is mainly expressed in the cytoplasm of HCC and adjacent liver tissues. Representative images of ZEB1 in HCC (E,F) and paired adjacent liver tissues (G,H). Positive expression of ZEB1 is mainly in cellular nuclei in HCC.

We detected the expression level of miR-203 in 73 pairs of HCC and matched adjacent noncancerous liver tissue samples by ISH. The results showed that miR-203 was mainly located in the cytoplasm of cells in the HCC and adjacent liver tissue samples. Its expression was decreased in 24.7% (18/73, low group) and increased or unchanged in 75.3% (55/73, high group) of the HCC samples compared with the adjacent liver tissue samples (Fig. 1). Furthermore, we analysed the relationships between miR-203 expression and clinicopathological factors in HCC. We found that decreased miR-203 expression was significantly associated with early recurrence (*P*=0.030). There were no significant correlations between miR-203 expression and other parameters, such as age, sex and tumor size (Table 1).

### Correlation between miR-203 and ZEB1 in early-stage HCC samples

**Figure 2:**
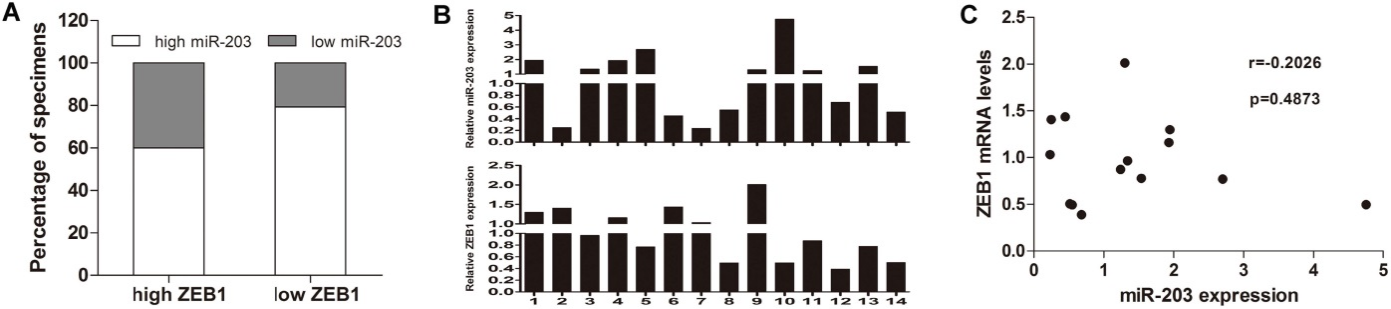
Association of ZEB1 expression with miR-203 expression in HCC samples. (A) Two-fold samples showed low miR-203 expression in the high ZEB1 expression group compared with the low ZEB1 expression group, But the difference was not statistically significant (*P*=0.122). (B) Expression of miR-203 and ZEB1 mRNA in 14 HCC tissues were determined by qRT-PCR. (C) The correlation between miR-203 and ZEB1 mRNA in HCC tissues (P=0.487).

To assess the correlation between miR-203 and ZEB1 in HCC, we used immunohistochemistry to measure ZEB1 expression in the same cohort used to examine the expression levels of miR-203. ZEB1 was detected mainly in the nucleus of HCC cells, and its frequency of expression was low. ZEB1 expression was increased in 20.5% (15/73, high group) and preserved in 79.5% (58/73, low group) of the samples (Fig. 1). No clinical factors, including age, sex, HBsAg status, microvascular invasion, tumor size, cirrhosis status, histological grade, AFP level, tumor encapsulation status and early recurrence status, were significantly associated with the upregulation of ZEB1 expression in HCC (Table 1). In the high ZEB1 expression group, 40% (6/15) of the samples showed low miR-203 expression. However, only 20.7% (12/58) of the samples in the low ZEB1 expression group exhibited low miR-203 expression. However, the difference was not statistically significant (*P*=0.122, Fig. 2A). The relationship between miR-203 and ZEB1 mRNA was also analysed in qRT-PCR (Fig. 2B). Results indicated that correlation coefficient was not significant enough (*P*=0.487, Fig. 2C).

### Downregulation of miR-203 expression correlates with a poor prognosis in patients with early-stage HCC

**Figure 3:**
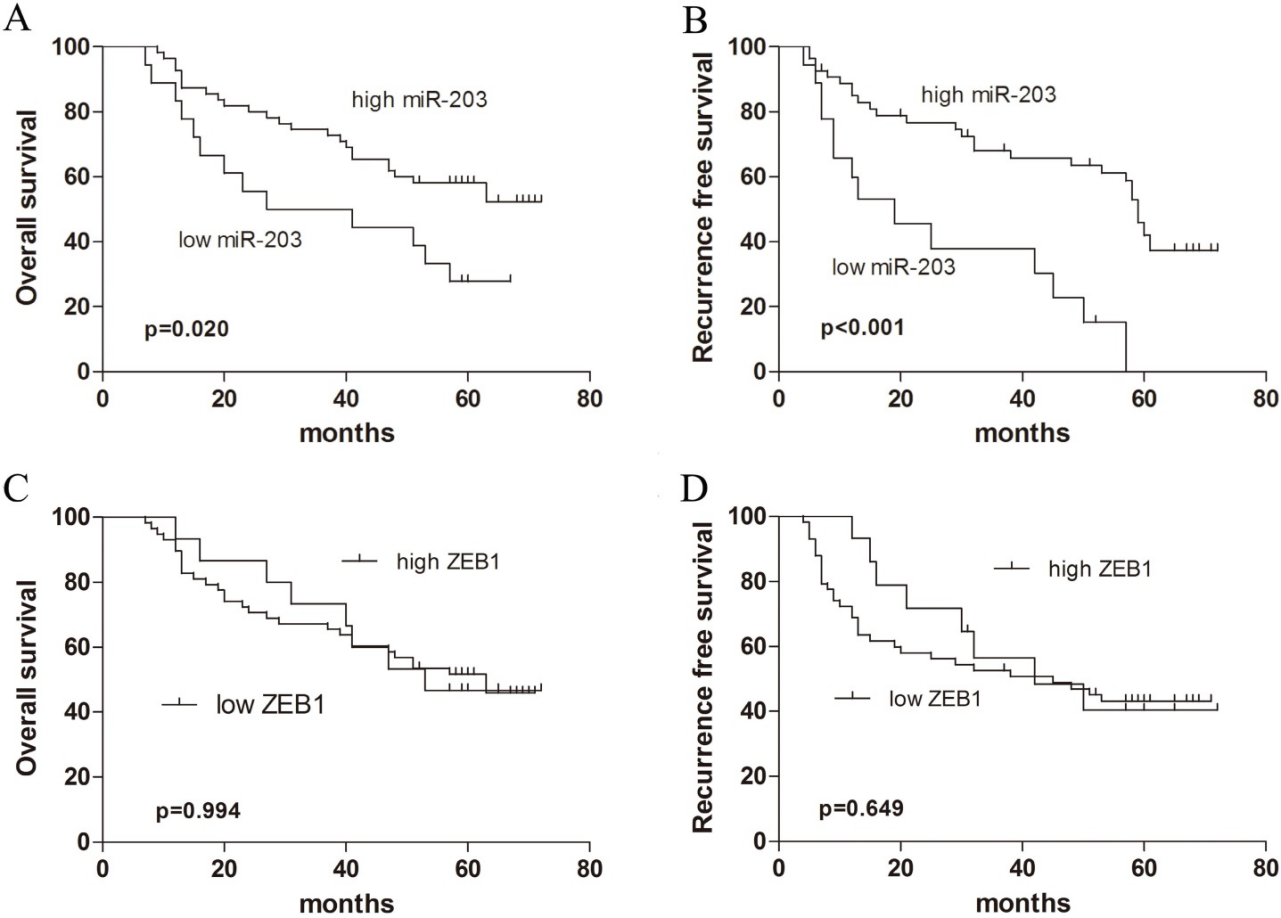
Survival curves in patients with HCC according to miR-203 and ZEB1 levels. (A) Overall survival curves in 73 patients with HCC according to miR-203 levels (*P*=0.020). (B) Recurrence-free survival curves in 73 patients with HCC according to miR-203 levels (*P*<0.001). (C) Overall survival curves in 73 patients with HCC according to ZEB1 levels (*P*=0.994). (D) Recurrence-free survival curves in 73 patients with HCC according to ZEB1 levels (*P*=0.649).

We examined whether the miR-203 and ZEB1 expression levels were correlated with the outcome of HCC after hepatectomy. Kaplan-Meier analysis revealed that HCC tissue samples with reduced expression of miR-203 correlated with shortened OS (Fig. 3A, *P*=0.020) and RFS (Fig. 3B, *P*<0.001). But ZEB1 expression was not correlated with survival of HCC patients (Fig. 3C, *P*=0.994 for OS, Fig. 3D, *P*=0.649 for RFS). Further Cox proportional hazards regression analysis was used to evaluate the association between miR-203 expression and prognosis. The final multivariate model revealed that miR-203 expression (*P*=0.036), AFP level (*P*=0.038) and microvascular invasion (*P*=0.015) were independent prognostic factors for OS in patients with early-stage HCC (Table 2). Moreover, on analyses of RFS, miR-203 (*P*=0.017), AFP level (*P*=0.000), tumor encapsulation (*P*=0.049) and tumor size (*P*=0.022) emerged as significant independent prognostic factors (Table 3).

**Figure 4:**
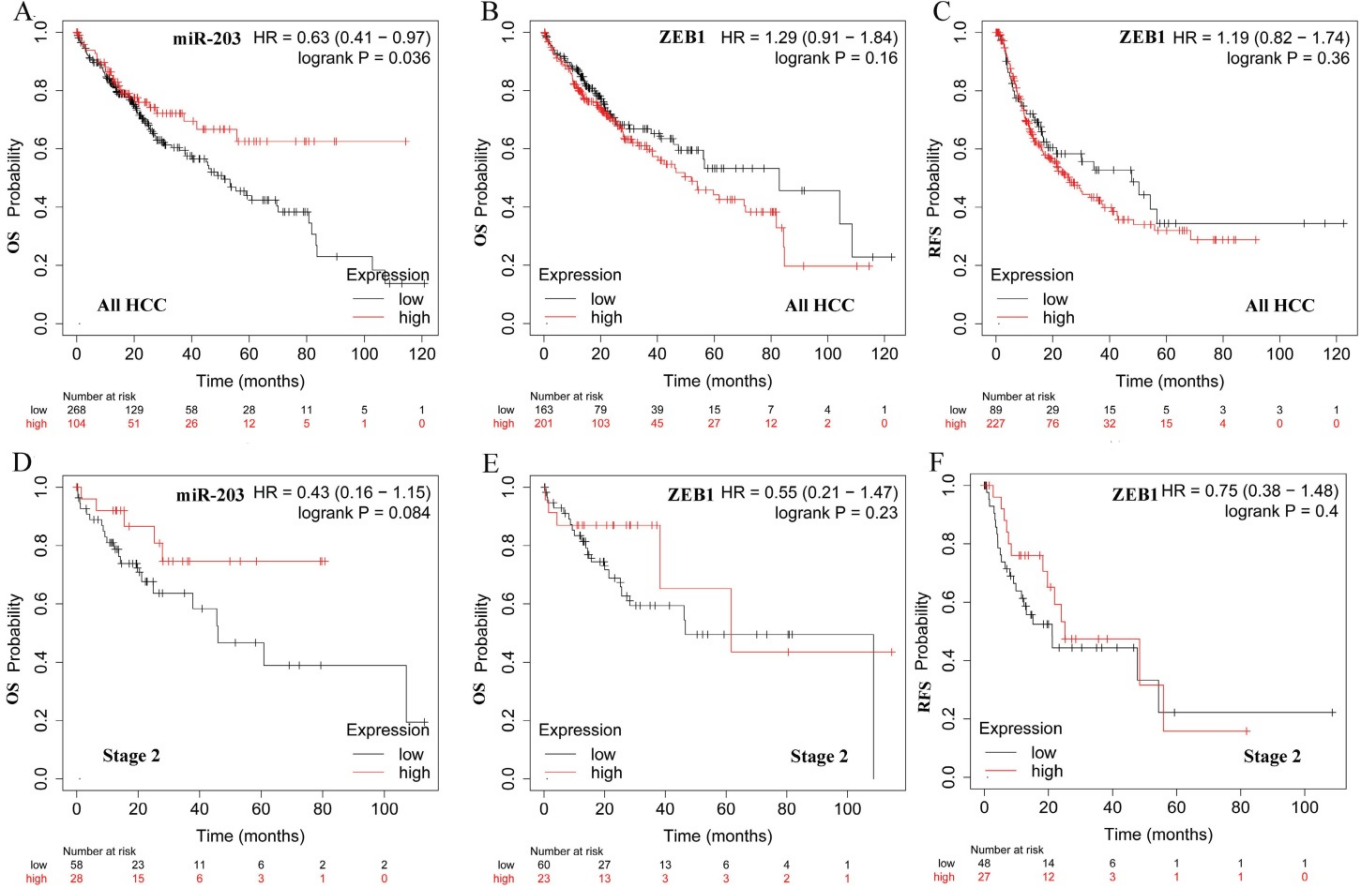
Prognostic significance of miR-203 and ZEB1 mRNA expression in HCC. The Kaplan-Meier plots were generated by Kaplan-Meier Plotter (http://kmplot.com). The effect of miR-203 expression level on the overall survival (OS) of all HCC patients(A). The effect of ZEB1 mRNA expression level on the overall survival (OS) (B) and recurrence-free survival (RFS) (C) of all HCC patients. The effect of miR-203 expression level on the overall survival (OS) of stage 2 HCC patients(D). The effect of ZEB1 mRNA expression level on the overall survival (OS) (E) and recurrence-free survival (RFS) (F) of stage 2 HCC patients.

We also examined the prognostic values of two factors in HCC patients in http://kmplot.com. The survival curves were shown in Fig. 4. We observed high miR -203 expression was significantly associated with better OS for all HCC patients (Fig. 4A, HR=0.63, 95% CI: 0.41-0.97, *P* =0.036). However, ZEB1 was not correlated with OS and RFS (Fig. 4B and C, HR=1.29, 95% CI: 0.91-1.84, *P* =0.16, HR=1.19, 95% CI: 0.82-1.74, *P* =0.36). In patients with early-stage HCC, the expression of miR-203 was modestly associated with OS (Fig. 4D, HR=0.43, 95% CI: 0.16-1.15, *P* =0.084). ZEB1 was not correlated with OS and RFS (Fig. 4E and F, HR=0.55, 95% CI: 0.21-1.47, *P* =0.23, HR=0.75, 95% CI: 0.38-1.48, *P* =0.4).

### MiR-203 inhibited the proliferation, migration and invasion of HCC cells *in vitro*

**Figure 5:**
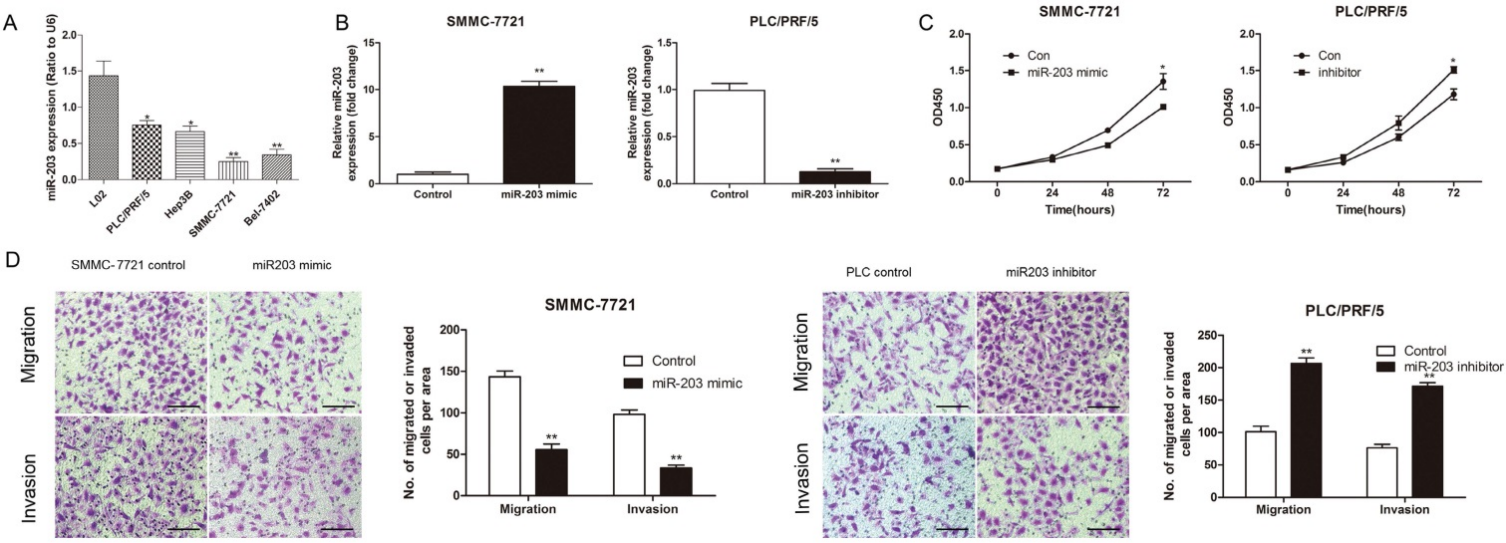
miR-203 inhibits the proliferation, migration and invasion of HCC cells *in vitro*. (A) Relative expression of miR-203 in different established HCC cell lines was determined by qRT-PCR. (B) Expression of miR-203 in SMMC-7721 and PLC/PRF/5 cells after transfection was determined by qRT-PCR. (C) CCK-8 assay was performed to detect cell proliferation, *P*<0.05. (D) Transwell assays were performed to detect cell migration and invasion, *P*<0.01, Scale bar = 100 μm.

To explore the function of miR-203 in HCC, we performed both gain- and loss-of-function experiments with the PLC/PRF/5 and SMMC-7721 cell lines, which had different levels of miR-203 expression (Fig. 5A). Successful modulation of the expression of miR-203 in each treated cell line was confirmed by qRT-PCR (Fig. 5B). A CCK-8 assay indicated that downregulation of miR-203 expression increased PLC/PRF/5 cell proliferation, whereas upregulation of miR-203 expression inhibited SMMC-7721 cell proliferation at 72 h after transfection (Fig. 5C). Transwell assays showed that PLC/PRF/5 cells transfected with a miR-203 inhibitor had a higher migratory ability and stronger invasive capacity than control cells, whereas upregulation of miR-203 expression could significantly suppress the migratory and invasive abilities of SMMC-7721 cells when compared with control cells (Fig. 5D). Therefore, miR-203 played suppressive roles in proliferation, migration, and invasion in HCC cells.

## Discussion

HCC is the second most lethal tumor, with a 5-year survival rate of 18%. As many as 70% of patients with a solitary tumor at an early stage (BCLC stage 0 or A) have tumor recurrence within 5 years after liver resection 24, 25. However, to date, it is still difficult to determine which individuals will have tumor recurrence after surgery, especially for patients with early-stage disease, and there are few effective therapies to reduce metastasis-related recurrence.

Recently, evidence has demonstrated that miRNAs are the major drivers of HCC metastasis at the post-transcriptional level 26, 27. We previously found that miR-203 expression was significantly associated with HCC recurrence and prognosis after LT according to microarray and real-time RT-PCR assays 9. Unlike hepatic resection, HCC recurrences after liver transplantation are all considered as metastasis-related recurrences because of complete liver removal 28. We considered that down-regulation of miR-203 may play an important role in HCC metastasis. In the present study, to further validate its prognostic value in early-stage HCC, we quantified miR-203 expression in an independent cohort comprising 73 HCC samples and examined the correlations between the expression of miR-203 and clinicopathological features, especially prognosis. To the best of our knowledge, this is the first time that the predictive value of miR-203 expression has been evaluated in HCC patients by using ISH. Our study showed that decreased miR-203 expression was associated with early HCC recurrence. Kaplan-Meier survival analyses revealed that HCC patients whose tumors displayed low expression of miR-203 had shorter RFS and OS than those whose tumors displayed high expression. Furthermore, multivariate Cox regression analysis indicated that low expression of miR-203 was an independent prognostic factor for poor survival in HCC patients after liver resection. Using a publicly available dataset (KM-plot), we also found that high miR-203 expression was significantly associated with better OS for HCC patients. *In vitro* experiments suggested that miR-203 inhibited HCC cell proliferation, migration and invasion.

A few studies have suggested that miR-203 serves as an HCC suppressor. Zheng et al. revealed that miR-203 downregulated IL-1β, Snail1 and Twist1 expression to inhibit EMT 29. Shao et al. noted that miR-203 increased cell radiosensitivity by directly targeting Bmi-1 30. These findings suggested that miR-203 might be employed as a therapeutic target in HCC. Recently, Chen et al. reported that miR-203 promoted liver regeneration after partial hepatectomy in cirrhotic rats 31. The two-sided effects of miR-203 support its potential application in clinically cirrhotic HCC patients.

Little is known regarding the causes of deregulated miR-203 expression in HCC. Furuta et al. found that miR-203 was silenced in HCC partly by promoter CpG hypermethylation 32. Two studies on pancreatic cancer reported that miR-203 was suppressed by ZEB1 19, 20. ZEB1 is master transcription factor that positively regulate invasion and metastasis by promoting EMT in cancer cells 33. In HCC, ZEB1 was reported to be significantly associated with tumor TNM stage, vascular invasion 14, 15. Patients expressing high levels of ZEB1 was associated with poorer prognosis. In the present study, we wanted to determine whether the ZEB1/miR-203 axis is involved in early stage HCC. We used immunohistochemistry to measure the protein expression levels of ZEB1 in the same cohort used to examine the expression levels of miR-203. Our results showed that no clinical factors, such as tumor size or prognosis, were significantly associated with the upregulation of ZEB1 expression. A potential explanation for the conflicting data could be that the patients in our study were all at a relatively early stage of disease (BCLC stage 0 or A). Sreekumar et al. demonstrated that the positive rate of ZEB1 was about 5% (1/19) in patients with TNM stage 1 or 2 HCC, while it was about 47.6% (10/21) in patients with TNM stage 3 HCC 16. Aberrant ZEB1 expression may not be an early event in hepatocarcinogenesis. To confirm our result, we used KM plotter database to investigate the prognostic value of ZEB1 mRNA in HCC. Our results indicated ZEB1 was not correlated with OS and RFS. We also measured the mRNA expression levels of ZEB1 in HCC tissues to explore the association between the expression of ZEB1 and miR-203. It was found that the correlation coefficient between miR-203 and ZEB1 in HCC tissues was not statistically significant.

Several limitations should be acknowledged in our study. First, this study is a retrospective single-centre study with limited sample size. Prospective multicentre clinical trials need to be performed to validate our results. Second, the underlying mechanisms of miR-203 in HCC remain unclear. Further studies need to be designed to elucidate the molecular mechanisms involving miR-203 in HCC metastasis.

In conclusion, we have demonstrated that low expression of miR-203 was related to tumor recurrence and poor survival in early-stage HCC patients who underwent curative surgery, but ZEB1 did not show any prognostic significance in our cohort. Additionally, functional studies indicated the anti-metastasis effect of miR-203 in HCC cells. Therefore, miR-203 could be regarded as a biomarker for predicting prognosis in early-stage HCC patients and might also be a potential molecular target for HCC therapy.

## Figures and Tables

**Table 1:** Correlation between miR-203 and ZEB1expression and HCC clinicopathological parameters.

		miR-203 expression			ZEB1 expression		
Parameters	Cases	Low	High	P value ^a^	Low	High	P value ^a^
Age(years)							
≤ 55	41	11	30	0.626	33	8	0.804
> 55	32	7	25		25	7	
Gender							
Male	59	15	44	1.000	49	10	0.146
Female	14	3	11		9	5	
HBsAg							
Positive	61	14	47	0.474	48	13	1.000
Negative	12	4	8		10	2	
MVI							
No	48	10	38	0.294	37	11	0.488
Yes	25	8	17		21	4	
Tumor size(cm)							
≤5	41	8	33	0.248	32	9	0.737
>5	32	10	22		26	6	
Cirrhosis							
Yes	61	16	45	0.718	48	13	1.000
No	12	2	10		10	2	
Histologic grade							
I+II	54	14	40	0.766	41	13	0.325
III	19	4	15		17	2	
AFP (ng/ml)							
≤400	47	12	35	0.816	36	11	0.417
>400	26	6	20		22	4	
Tumor encapsulation							
Presence	37	9	28	0.947	27	10	0.165
Absence	36	9	27		31	5	
Recurrence							
Yes	40	14	26	0.030^*^	32	8	0.898
No	33	4	29		26	7	

AFP alpha-fetoprotein, MVI microvascular invasion; ^a^ Statistical analysis were performed with Chi-square test or Fisher exact test. ^*^ statistically significant difference.

**Table 2:** Univariate and Multivariate Analyses of factors associated with Overall Survival of 73 Patients with early-stage HCC.

Characteristic	HR	95%CI	P-value
Univariate analysis			
Age (>55)	1.365	0.716-2.601	0.345
Gender (female)	1.224	0.791-1.896	0.365
HBsAg (Positive)	0.837	0.554-1.264	0.398
Tumor size (>5 cm)	2.072	1.080-3.975	0.028^*^
Cirrhosis (Yes)	0.838	0.554-1.267	0.402
Histologic grade (III)	1.374	0.627-3.012	0.428
AFP ≤400 (ng/ml)	0.518	0.270-0.993	0.048^*^
MVI (Yes)	2.239	1.170-4.286	0.015^*^
Tumor encapsulation (Presence)	0.865	0.454-1.650	0.660
ZEB1 (High)	1.089	0.527-2.251	0.818
miR-203 (High)	0.460	0.233-0.905	0.024^*^
Multivariable analysis			
miR-203 (High)	0.484	0.246-0.954	0.036^*^
AFP ≤400 (ng/ml)	0.502	0.261-0.964	0.038^*^
MVI (Yes)	2.238	1.167-4.291	0.015^*^

AFP alpha-fetoprotein, MVI microvascular invasion, HR hazard ratio; CI confidence interval. ^*^ statistically significant difference

**Table 3:** Univariate and Multivariate Analyses of factors associated with recurrence-free survival of 73 Patients with early-stage HCC.

Characteristic	HR	95%CI	P-value
Univariate analysis			
Age (>55)	0.958	0.513-1.786	0.892
Gender (female)	1.310	0.848-2.023	0.224
HBsAg (Positive)	1.128	0.706-1.803	0.615
Tumor size (>5 cm)	2.258	1.207-4.226	0.011^*^
Cirrhosis (Yes)	0.807	0.337-1.933	0.630
Histologic grade (III)	0.932	0.465-1.866	0.842
AFP ≤400 (ng/ml)	0.307	0.164-0.575	0.000^*^
MVI (Yes)	1.675	0.891-3.149	0.109
Tumor encapsulation (Presence)	0.537	0.285-1.012	0.054
ZEB1 (High)	0.744	0.354-1.565	0.436
miR-203 (High)	0.431	0.224-0.832	0.012^*^
Multivariable analysis			
miR-203 (High)	0.438	0.222-0.861	0.017^*^
AFP ≤400 (ng/ml)	0.300	0.158-0.567	0.000^*^
Tumor size (>5 cm)	2.104	1.113-3.980	0.022^*^
Tumor encapsulation (Presence)	0.521	0.272-0.997	0.049^*^

## Abbreviations

ZEB1: Zinc finger E-box binding homeobox 1
HCC: hepatocellular carcinoma
BCLC: the Barcelona Clinic Liver Cancer
EMT: epithelial-to-mesenchymal transition
AFP: alpha-fetoprotein
MVI: microvascular invasion
RFS: recurrence-free survival
OS: overall survival
IHC: immunohistochemistry
ISH: *in situ* hybridization
GEO: Gene Expression Omnibus
TCGA: The Cancer Genome Atlas
